# Preparation for Training in the Heat Season: Key Considerations for Heat Injury Risk Prevention and Mitigation

**Published:** 2025-07-20

**Authors:** Gabrielle E. W. Giersch

**Affiliations:** Thermal and Mountain Medicine Division, U.S. Army Research Institute of Environmental Medicine

Training in the heat substantially increases risk of exertional heat illness, a risk accepted by military units due to the necessity of training in environments similar to those where warfighters may be deployed.^1^ Previous work has shown that foot marches (i.e., ruck marches) and timed runs are the activities with highest heat-related illness prevalence.^2^ Severe heat-related illnesses, such as exertional heat stroke (EHS), can lead to long recovery times and end organ damage, and constitute a significant threat to force lethality and detriments to deployability.^1,3^ While research and guidelines for appropriate conduct of service member activities in hot environments are updated regularly, over 2,500 warfighters continue to be affected by heat-related illnesses annually.^4^

Subject matter experts at the U.S. Army Research Institute of Environmental Medicine (USARIEM) conduct research to both prevent heat-related illness and enhance physical performance in the heat, with a particular emphasis on the needs of the warfighter. USARIEM research has led to key developments for enhancing performance along with prevention and treatment strategies for heat-related illnesses.^5-8^ The cumulative knowledge from this work leads to guidelines published (among others) in *Technical Bulletin, Medical: Heat Stress Control and Casualty Management*, which provides resources—including fluid prescription guidelines and work-to-rest ratios—for all flag conditions, as well as details on risk factors to prevent the development of heat illness.^9^

This editorial summarizes the results and conclusions of recent biomedical research on performance and injury risk in the heat, to provide practical considerations for warfighters training in the heat, both for individual warfighters as well as leaders who plan training activities. This editorial describes recent evidence about individualized factors that may influence risk of developing a heat-related illness and strategies to prepare for training in the heat.

## Physiology of Exercise Heat Stress and Illness

Thermoregulation during physical activity in the heat has been extensively reviewed in the literature.^9-12^ Environmental heat stress, particularly with exercise, increases risk of heat-related illness.^3,13-15^ During exercise heat stress, working skeletal muscle produces heat, with the heart rate elevating to increase cardiac output to accommodate the needs of the working skeletal muscle, as well as the skin, for heat dissipation. The primary heat dissipation mechanisms during exercise are evaporation—of sweat from the surface area of the skin—and convection—from air flow over the skin surface in combination with increased skin blood flow.^16-18^

Exertional heat illnesses constitute a spectrum, ranging in severity from heat exhaustion, defined as an inability to continue exercise in the heat, to heat injury, which is defined as heat exhaustion with evidence of end organ damage, and EHS, which is elevated body temperature with altered mental status.^3,19^ Altered mental status associated with EHS can also range from change in walking or running gait, to slurred speech, to loss of consciousness.^20^ Organ damage is also prevalent in EHS cases, with recent evidence suggesting a possible sex difference in level of end organ damage despite apparent similar EHS severity.^21^ Importantly, classic heat stroke differs from EHS: Classic heat stroke more often affects older individuals during heat waves, associated with hot, dry skin and no physical activity, whereas EHS casualties occur more often in younger people sweating due to physical activity.^19^

## Preparation Strategies and Considerations

Successful prevention of heat-related illness requires preparation well in advance of heat season; these preparations can include heat acclimatization and increased fitness levels for at least 1 month prior. Heat acclimatization is the most effective means for not only heat stress preparation and decreasing risk of heat-related illnesses, but enhancing performance as well. Heat acclimatization has been extensively investigated, with various protocols demonstrating enhanced performance and thermoregulatory function during heat stress.^22^ Heat acclimatization is the process of repeatedly exposing the body to heat stress in a controlled manner, with incremental and gradual increases in exercise intensity to allow beneficial adaptations.^23-25^ The primary adaptations of heat acclimatization include decreased resting and exercise body temperatures, lower exercise heart rate, increased sweating rate, and increased physical performance capacity.^23^ The process can occur over as little as 4-8 days with longer durations prescribed (e.g., 10-21 days) to ensure greatest possible adaptation.^23-25^

While heat acclimatization is the most effective preparation strategy, increased fitness from training in cool and temperate environments can also enhance performance during training in the heat, and may reduce risk of heat-related illness.^26^ Individuals with higher fitness statuses are thought to have preliminary heat adaptations (i.e., partial acclimatization) that are likely due to increases in body temperature during extended exercise, which allow milder adaptations.^10^ Endurance training (e.g., running) has been observed to provide the most beneficial adaptations to heat stress.^10^ Once training and acclimatization have been achieved, maintenance (i.e., continued exercise or heat exposure) is important to prevent diminishment of those adaptations.^27^

A key consideration for heat training preparation is awareness of biological and physiological factors that increase risk of heat illness.^28^ Recent USARIEM research evaluating risk factors for heat illness among groups and individuals has found that higher body mass index (BMI), which is associated with a lower body surface area to mass ratio (BSA:mass), increases individual risk for EHS.^7,8^ Every 1 unit increase in BMI led to a 3% increase in relative EHS risk.^7^ Additional work from the Uniformed Services University of the Health Sciences (USUHS) confirmed this effect of BMI.

Research at USUHS also observed increased risk of heat-related illnesses in conjunction with both upper and lower respiratory infections.^29^ Lower respiratory infections (e.g., influenza, bronchitis) have strong effects on EHS risk. Adequate recovery from respiratory infections prior to participation in heat training is particularly important for reducing risk of developing an exertional heat-related illness.^29^

Decades of research into prevention of heat-related illnesses and EHS have identified many biological, physiological, behavioral, and environmental risk factors, summarized in the **Table**. This evidence can inform decision-making for unit leaders as well as individuals, for appropriate preparation, adjustment, modification, and completion of training exercises and minimization of heat training casualties.

**TABLE.** Commonly Cited Risk Factors for Heat-related Illnesses

Risk Factor	Supporting Evidence
Biological	
Sex	Evidence for sex as a risk factor is mixed and may depend upon illness severity or sociological factors. A case-control analysis that directly evaluated sex and EHS risk found no sex influence, but studies investigating prevalence by heat illness severity show conflicting findings: Earlier findings show women at a greater EHS risk, while more recent findings show men at greater EHS risk and women at greater heat exhaustion risk. If sex difference for heat-related illness risk exists, it may not be related to physiological but, instead, cultural or practical factors (for example, anecdotal reports from allied militaries state that some women may voluntary dehydrate due to limited latrine access, but this remains to be investigated).^7,28,29,38^
Race and ethnicity	Non-Hispanic Black individuals appear to be at greater risk for heat illness, but physiological factors are unclear. Individuals with sickle cell trait (more prevalent in non-Hispanic Black individuals) have increased risk of heat illness, but race has been shown previously to increase risk independent of sickle cell trait status.^39^
BMI	Each 1 unit increase in BMI is associated with 3% increase in relative EHS risk, along with higher risk of more minor heat illness.^7^
Physiological	
Acclimatization	Gradual adaptation to heat decreases heat illness risk.^3,23^
Poor physical fitness	Poorer fitness status increases risk for heat-related illness.^10,26^
Respiratory infection	Respiratory infections affect risk for all forms of heat illness.^29^
Behavioral	
Hydration	Hydration before and during activity can reduce risk of heat-related illness. Dehydration increases body temperature during training in heat and increases rate of body temperature rise. Dehydration may influence performance and ability to continue activity. While starting an activity well-hydrated and remaining hydrated help reduce heat illnesses risk, hydration alone does not protect individuals from heat illness. Hydration is only 1 of many risk mitigation measures. Avoiding alcohol 48-72 hours prior to training can help maintain appropriate hydration, as alcohol acts as a diuretic.^40^ Alcohol can also induce fatigue or alcohol-related symptomology during training.^41-43^
Supplementation and medication	Certain medications and supplements (e.g., stimulants) can increase body temperature, with potential increased heat illness risk, but no direct risk analysis has been conducted. Energy drinks with high amounts of caffeine and supplements are not regulated by the FDA and may contain harmful ingredients that can exacerbate elevated body temperature during exercise and heat stress, and may may influence heat-related illness risk.^44-46^
Sleep	Although often characterized as a risk factor, there is limited evidence to support the assertion that sleep deprivation is a risk factor for heat illness; no physiological mechanism is known. Adequate sleep may help ensure best preparation for training and thereby aid risk reduction for heat-related illness.^47^
Personal motivation	Individuals inclined to exert themselves harder (e.g., candidates for promotion or elite level training) are at increased risk if inclined to ignore or minimize accepted physiological or perceptual indicators of fatigue or overheating.^48^
Environmental	
Weather	Radiant heat load and humidity are factors in high WBGT, an aggregate temperature measure incorporated in DOD guidelines, which allow for flexible training times (e.g., earlier in the day), reduced radiant heat loads, work-to-rest ratios, and fluid replacement guidelines. Other weather factors such as heat index or UTCI have been proposed, but the most widely accessible, with greatest data, is WBGT.^49-51^
Load carriage	Higher weight loads intensify body heat production and thereby increase physical burden to diffuse that heat.^9^
Clothing	Greater clothing insulation (e.g., Army combat uniforms) increase thermal load of an activity by decreasing capacity for heat dissipation.^9^

Abbreviations: EHS, exertional heat stroke; BMI, body mass index; FDA, Food and Drug Administration; WBGT, wet-bulb globe temperature; DOD, Department of Defense; UTCI, Universal Thermal Climate Index.

## Mitigation Measures

The ability to cool and diffuse heat before or during training activities is paramount for decreasing risk. The Arm Immersion Cooling System (AICS) is a method used to cool service members prior to, or during, activities of greatest risk.^6^ AICS is most effective if the protocol is properly adhered to, specifically resting the arms (wrists to elbows) in cool ice water for 3-5 minutes.^6^

In addition to cooling before and during activity, readily available treatment remedies are vital for ensuring prompt recognition, arrest, and recovery of heat illness casualties. Cooling modalities range in both practicality and effectiveness. Ice water immersion is the ‘gold standard’ for cooling a heat casualty as quickly as possible^30^ but requires a significant amount of ice and water, which can only be used for 1 casualty. Iced sheets is a method with greater practicality but slightly less cooling efficacy. While cooling rates for iced sheets are lower, if used properly—rotated *at least* every 3 minutes—they are an effective means of cooling heat casualties.^5^ If iced sheets are not rotated frequently enough, they can trap heat and exacerbate elevated body temperatures.^5^

## Injury Identification and Recovery

Prompt identification of a heat casualty can drastically reduce long-term complications, as the amount of time hyperthermic appears to be indicative of the level of damage from the heat illness.^31^ Knowing signs and symptoms including altered mental status (AMS), including confusion, change in walking or running gait, slurred speech, and loss of consciousness, are primary examples of AMS.^32,33^

Early identification and initiation of cooling can enhance recovery and return-to-duty (RTD) for heat-related illnesses.^34^ RTD and recovery from a heat-related illness vary depending on illness severity.^34^ Many heat exhaustion cases can return to duty and training the following day with appropriate guidance and hydration, while EHS cases can require many weeks.^35,36^

EHS cases can range in severity as well, largely dependent on prompt detection, cooling onset, and pre-hospital care.^34,35^ Organ damage is common in EHS, with some individuals necessitating organ transplants (usually liver or kidney). Army Regulation 40-501 governs RTD for soldiers and specifies minimum EHS recovery of 10 weeks.^37^

It is commonly suggested that becoming a heat illness casualty, particularly with EHS, puts an individual at an increased risk for subsequent heat illness, but there is limited research to support this assertion. Full and optimal recovery leads to complete RTD, likely without increased risk for future heat illness.^35^

## Enhanced Prevention and Mitigation Strategies

The prevalence and impacts of heat-related illnesses, on individual warfighter lethality and medical readiness, make continued research efforts for enhanced performance, prevention, recovery, and RTD for heat-related illnesses of continuing importance. Current mitigation efforts at USARIEM include identification of bio-markers of recovery from heat-related illnesses for enhancing RTD, development of practical heat acclimatization strategies that do not require a laboratory nor hot environment, evaluation of inter-individual variability in heat stress as well as physiological and biomarker responses, and enhanced prevention efforts for both individuals and units.

With most military training installations located in the southeastern U.S., training in the heat is a necessity for the U.S. military. It is critical to ensure appropriate preparation and education of risk factors and signs of heat illness and injury for both individual service members and leadership, for their recognition and mitigation of these risks during training events. While heat-related illnesses, including EHS, are not 100% preventable, mitigation of risk factors and adequate preparation can reduce as many as possible, to optimize the health and lethality of our warfighters—both during training and in battlefield environments.
